# When the water heats up, brown trout pay the price

**DOI:** 10.1093/conphys/coaf072

**Published:** 2025-10-14

**Authors:** Md Fazle Rabbe

**Affiliations:** Department of Zoology, University of Dhaka, Dhaka 1000, Bangladesh

As climate change raises river temperatures, a silent struggle is going on beneath the surface. Brown trout (*Salmo trutta*), a temperature-sensitive fish species, is facing a physiological battle that could change their future. A recent study conducted in Austria reveals that even short-term exposure to warmer water can dramatically affect the way brown trout use energy, respond to stress, and grow (Hampuwo *et al.*, 2025). In Austria, some summer streams are already surpassing 20°C—a critical threshold for brown trout. Researchers wanted to determine how this warming affects the fish at multiple levels, from their genes to their growth. What they discovered is a cascade of biological changes triggered by temperature.

Brown trout, a vulnerable species of the salmon family, are sensitive to changes in water temperature and quality, making them important indicators of ecosystem health. Considering the narrow temperature limits in which the species can survive, it is estimated that they will be unable to survive in 64% of the European rivers by 2080.

To investigate how brown trout responded to different temperatures, Hampuwo and team raised groups of young brown trout (Fig. 1) in two types of stream tanks. They used 250 fish dividing them between four artificial stream channels. Two streams were kept at a constant 9°C, while two were warmed gradually to 20°C over a week and held there for three weeks. When the trout were placed in the warmer water, it triggered a stress reaction in their bodies. This caused certain helpful genes to become more active. These genes are known to maintain cell function and keep them working properly when under stress. As the heat stress continued, trout began burning through their energy reserves to cope. The liver, where fish store important energy sources like sugars and fats, showed a big drop in those reserves. Even the fish’s overall energy levels, a kind of ‘fuel gauge’ for their health, went down.

**Figure 1:**
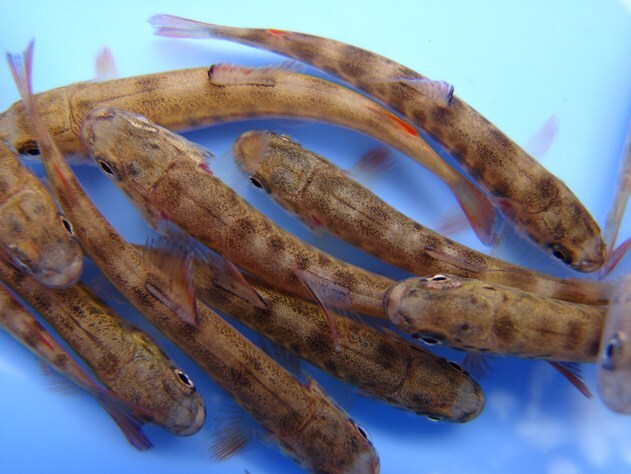
Young brown trout. Image credit: Stefan Strobl

The team checked the fish at three different times (before, during, and after heat exposure). They measured fish growth, collected liver tissue and tested for gene activity and energy metabolite levels. Although nearly all the heat-stressed trout (98%) survived, they grew 43% less than their cool-water cousins during the experiment. The team also found 70% reduction in abdominal fat in the heat-stressed fish, indicating that they were breaking down their stored fat to cope with the heat. These changes highlight the hidden energy toll that rising water temperatures can take.

This study shows that even just three weeks at 20°C is enough to alter the physiology of brown trout. These findings raise important questions for conservation and fishery management. To protect species like brown trout, we will need to combine climate action with smart, science-based conservation efforts.

## Notes

**Editor:** Ashleigh Donaldson
